# Alexithymia, Metacognition, and Theory of Mind in Children and Preadolescents With Migraine Without Aura (MWoA): A Case-Control Study

**DOI:** 10.3389/fneur.2019.00774

**Published:** 2019-07-17

**Authors:** Giulia Natalucci, Noemi Faedda, Alessia Quinzi, Danilo Alunni Fegatelli, Annarita Vestri, Giada Turturo, Paola Verdecchia, Benedetta Bellini, Chiara Pirisi, Dario Calderoni, Flavia Giannotti, Rita Cerutti, Teodosio Giacolini, Vincenzo Guidetti

**Affiliations:** ^1^Section of Child and Adolescent Neuropsychiatry, Department of Human Neuroscience, Sapienza University of Rome, Rome, Italy; ^2^Behavioural Neuroscience, Section of Child and Adolescent Neuropsychiatry, Department of Human Neuroscience, Sapienza University of Rome, Rome, Italy; ^3^Department of Public Health and Infectious Diseases, Sapienza University of Rome, Rome, Italy; ^4^Department of Maternal and Child Health and Urological Science, Sapienza University of Rome, Rome, Italy; ^5^Department of Dynamic and Clinical Psychology, Sapienza University of Rome, Rome, Italy

**Keywords:** alexithymia, metacognition, theory of mind, children, pre-adolescents, migraine without aura, MWoA

## Abstract

**Background:** Some studies have demonstrated the high impact of headache and migraine in several areas of children and adolescents' life. In recent years, there has been an increase in scientific interest in the relationship between migraine and emotional regulation, investigating the possible consequences of emotional dysregulation on physical and mental health. While some studies have been carried out on the relationship between alexithymia and headache or migraine (especially in adults), no data exist on relationship between Theory of Mind (TOM), metacognition, and alexithymia in children and adolescents with migraine.

**Methods:** Children with diagnosis of migraine without aura (MWoA) (36 males and 34 females) were compared to a healthy control group (31 males and 39 females). The age range was from 8 to 13 years in both groups. All children completed the *Alexithymia Questionnaire for Children* (AQC) for the assessment of alexithymia levels and the Domain of Social Perception included in the *NEPSY-II* to evaluate levels of TOM. Metacognitive development was evaluated with *Io e La Mia Mente* for children aged between 8 and 10 years and with *Metacognitions Questionnaire for Children* (*MCQ-C*) for children from 11 to 13.

**Results:** There were no differences between children with MWoA and the control group in metacognitive abilities; only in the subscale “Negative Meta Worrying” of *MCQ-C* girls scored higher than boys, regardless of the group they were part of. Also, in the *NEPSY-II* subscale there were no statistically significant differences between the two groups. Children with MWoA scored higher in the *AQC* subscales “Difficulty Identifying Feelings” and “Difficulty Describing Feelings” than controls. Moreover, children between 8 and 10 years statistically differed from older children in “Difficulty Identifying Feelings” and in Total Score.

**Conclusion:** Our data suggest that children with MWoA have no metacognitive and TOM problems compared to a healthy group. The experimental group showed higher traits of alexithymia, confirming what suggested by other studies in the literature. Future research will have to focus on migraine with aura and tension-type headache to evaluate any differences with children with MWoA.

## Introduction

Headache is one of the most common neurological symptoms reported in childhood and adolescence and constitutes a serious health problem that may lead to high levels of school absenteeism and to impairment in several other areas (1, 2). Furthermore, it is a disorder associated with numerous comorbid conditions that can determine a negative impact on the quality of life and individual well-being (3, 4). For example, headache is often associated to emotional and behavioral problems, especially internalizing disorders such as depression and anxiety, medical problems like asthma, allergies, sleep disorders, obesity, etc. (5). These factors can negatively affect child and family alike interfering in daily activities, social interaction, and negatively affecting school performance (6). Recently, many researchers are focusing their interest on the relationship between headache or migraine and emotional regulation and, in particular, they are trying to explore the possible consequences of emotional dysregulation on physical and mental health (7, 8). An interesting research line concerns the relationship between alexithymia and headache. Alexithymia refers to a difficulty in identifying, verbalizing and describing feelings, to a reduced level of emotional experience and to a limited imagination (9). Starting from the 1990s, there had been accumulating evidence of alexithymic characteristics in adult patients with primary headache (10–12). However, little research has been conducted on the relationship between alexithymia and primary headache in developmental age, and no study has investigated the cognitive and socio-relational aspects associated with this disorder (13). We decided to investigate a possible connection between alexithymia and metacognitive abilities or Theory Of Mind (TOM) disfunctions in subjects with primary headache. In fact, metacognition refers to the perception and regulation of emotions (in oneself and in other people), to their development, to their receiving causal attribution and meaning (14). Cognitive monitoring and the ability to mentally represent one's own and others' mental state can be considered fundamental pre-requisites for the recognition and the autonomous regulation of emotions (15). Currently only few studies have addressed the association between headache or migraine and impairment on metacognitive or TOM abilities in children and adolescents (16).

For these reasons, we picked as primary objective of this case-control study the comparison of the prevalence of alexithymia and deficit in TOM and metacognition abilities in pediatric patients with migraine without aura (MWoA) and in healthy control subjects. Our secondary objective was the comparison of test scores between children and pre-adolescents and between males and females. Finally, we intended to highlight any significant results within the experimental Group (EG).

## Methods

### Participants

The study involved a total of 140 subjects divided into two groups: EG consisted of 70 subjects (36 males and 34 females) aged 8 to 13 years (mean age 10.18, SD 1.55), attending the Center for the diagnosis and treatment of Juvenile Headache located in the Department of Pediatrics of the Policlinico Umberto I Hospital in Rome. These children and pre-adolescents had previously received a diagnosis of MWoA according to The International Classification of Headache Disorders 3rd edition beta version (ICHD-3B) (17). The inclusion criteria were the presence of an established diagnosis of MWoA and the absence of any psychiatric disorders or a diagnosis of a secondary headache. Table 1 presents the basic demographic data for the two groups. Table 2 lists the key characteristics of the EG.

**Table 1:** Demographic data.

	**Age**		**Total**	**Gender**		**Total**
	**8–10**	**11–13**		**M**	**F**	***N***
EG	38 (54.3%)	32 (45.7%)	70	36 (51.4%)	34 (48.6%)	70
CG	33 (47.1%)	37 (52.9%)	70	31 (44.3%)	39 (55.7%)	70
	71 (50.7%)	69 (49.3%)		67 (47.9%)	73 (52.1%)	140

*EG, Experimental Group, CG, Control Group*.

**Table 2:** Key characteristics of the experimental group.

Characteristics			*N*	
Diagnosis	Migraine without aura (MWoA)		70	
Time since the beginning of pharmacologic treatmen.	≥6 months		29 (41.4%)	
	<6 months >1 year		17 (24.3%)	
	<1 year		24 (34.3%)	
Years from the onset	≥3 years		34 (48.6%)	
	<3 years		36 (51.4%)	
Associated symptoms	Photophobia and/or phonophobia		30 (42.8%)	
	Photophobia and/or phonophobia + Vomiting/nausea/pallor/dizziness		40 (57.2%)	
Therapy	Acute treatment	43 (61.3%)	Paracetamol	35 (50%)
			Ibuprofen	8 (11.4%)
	Preventive treatment	13 (18.7%)	Amitriptyline	6 (8.6%)
			Flunarizine	2 (2.9%)
			Cinnarizine	5 (7.1%)
	Acute Treatment added to integrator 14 (20%)		Pineal tens	14 (20%)
Frequency of episodes	>1 a week		59 (84.3%)	
	2–3 a week		9 (12.8%)	
	<3 a week		2 (2.9%)	
Intensity	Low		57 (81.4%)	
	Medium		10 (14.3%)	
	Severe		3 (4.3%)	

The control group (CG) consisted of 70 healthy subjects (31 males and 39 females) (mean age 10.57 SD 1.66), of age and gender matched to the EG. The exclusion criteria were the presence of a medical history of primary and secondary headaches and any psychiatric disease. Both parents of all the children signed a written consent before their inclusion in the research. The EG clinical information were collected through both the headache diary and the clinical record.

Written informed consent was obtained from all parent's subjects. The study was conformed to the ethical guidelines of the 1975 Declaration of Helsinki and was approved by the Ethical Committee of Policlinico Umberto I, Sapienza University of Rome (Prot. N 336/19).

### Measures

#### Alexithymia

Alexithymia was assessed by the validated Italian version (18) of the *Alexithymia Questionnaire for Children* (AQC) (19), a simplified form of the original adult questionnaire for alexithymia developed by Bagby et al. (20) (*Toronto Alexithymia Scale*, TAS-20). Consistent with the *TAS-20*, this 20-item self-rating questionnaire measures the following three factors: Difficulty Identifying Feelings (DIF), Difficulty Describing Feelings (DDF), externally-oriented thinking (EOT). Responses to each item are given using a 3-point Likert scale with a score ranging from 0 (not true), 1 (a bit true) to 2 (true). This different approach from the traditional 5-point scale (used for the *TAS-20*) was used to simplify the response scale for children and to provide clear verbal labels for each answer category. DIF is an index of respondents' difficulty in identifying an experience as an affective state: seven items (1, 3, 6, 7, 9, 13, 14) assess the ability to identify feelings and to distinguish them from the somatic sensation that accompanies emotional arousal. DDF consists of five items (2, 4, 11, 12, 17) that assess the capacity to name and describe feelings to other people. EOT consists of eight items (5, 8, 10, 15, 16, 18–20) assessing EOT. Finally, there is the Total Score obtained by the sum of all scores: a high total score indicates greater alexithymic tendencies. Based on other research, this test can be administrated to children 8 years of age and older (21, 22).

#### Metacognition

To measure the level of metacognitive development of subjects aged between 8 and 10 years, the questionnaire “*Io e la mia mente”* (IeLMM) was administered (23). This questionnaire allows to assess the child's basal metacognitive level. The items stimulate a reflection on the mental world and on the strategies used for memory and learning tasks. All the items are accompanied by a graphic representation that simplifies the content and helps with the compilation of the test. It is a 15-item self-rating questionnaire with multiple choice answers and it can be administered individually or collectively, with no time limits. The final score is obtained by adding the score of correct answers; higher scores indicate greater metacognitive abilities.

To measure the level of metacognitive development of subjects aged between 11 and 13 years the Italian validated version (24) of the “*Metacognitions Questionnaire for Children*” (MCQ-C) (25) was administered. The adaptation by Bacow et al. (25) consists in simplifying the language of some items for use with young children. It allows to separate functional vs. dysfunctional forms of the meta-cognitive processes and it comprises 22 items and five subscales: Positive Meta-Worry (PMW); Negative Meta-Worry (NMW); Superstition, Punishment and Responsibility (SPR); Cognitive Monitoring (CM). Participants respond using a 4-point Likert scale indicating the agreement with each statement (from 1 “do not agree to 4” agree very much). The total score is considered a general measure of metacognitive awareness and processes and it range from 22 to 88, with higher scores indicating greater negative metacognitive activity. Since the *MCQ-C* test in Italy had already been used on children aged 11 and over (24), according to our knowledge, we preferred to use a different test for younger children, separating younger children from pre-adolescents. Furthermore, we noticed that a few 8-year-olds had some difficulty in understanding the questions of the *MCQ-C* and therefore we opted for this alternative method.

#### Theory of Mind

The level of the development of the theory of mind was assessed by two sub-tests from the Domain of Social Perception included in the *NEPSY-II* (26, 27), a battery for neuropsychological assessment in children aged between 3 and 16 years. These two tests investigate the ability to interpret the intentions and point of view of others and the ability to understand how these affect people's behavior. It is divided in two parts: *Part A* (Verbal Task) assesses the ability to understand mental constructs (such as beliefs, intentions, emotions, fantasy, and fiction) as well as the ability to understand that others have their thoughts, ideas, feelings that may differ from the observer's. It includes 15 items (questions on stories and pictures that require an understanding of the other's point of view). *Part B* (Contextual Task) assesses the ability to understand how emotions are connected to a social context and to recognize the appropriate state of mind. It includes eight items (a picture representing a social context and four alternative photos that represent the possible mood of the protagonist). Each item can get a score of 0 or 1 depending on the accuracy of the answer and the total is calculated by adding the scores of all the items.

### Statistical Analysis

Categorical data were recorded as absolute frequencies and percentages. Sub-scale scores were summarized in terms of median and interquartile ranges. Since the data are not normally distributed, the study employed Mann-Whitney and Kruskal-Wallis Test to find out differences between the groups at different tests (*IeLMM, MCQ, AQC, TOM*). Spearman correlation test was used to assess the correlation between sub-scale scores. The data were analyzed with the free statistical software R, version 3.5.1 (https://www.r-project.org/), and for each statistical analysis were considered significant *P* < 0.05.

## Results

The scores tests of the EG and the CG were compared, then, were compared males and females regardless of group and age and finally, children and pre-adolescents (8–10 vs. 11–13 years). We also study features within the EG. Initially, several clinical variables were collected in the migraine patients, with the idea of looking for a possible association between the psychological measures detected and the headache characteristics. However, the variables “frequency” (≤ 2 weeks/between 2 and 3 weeks/>3 weeks), “intensity” (low/medium/severe), and “therapy” were excluded from the analysis because they were characterized by a strong heterogeneity and it was not possible to make a comparison (as shown in Table 2). Instead, the variables “years from onset,” (more or less than 3 years), “associated symptoms” (presence of other symptoms besides phonophobia and photophobia) and “Time since the beginning of pharmacologic treatment” (≤6 months–between 6 months and 1 year, >1 year) have been studied.

**Figure 1:**
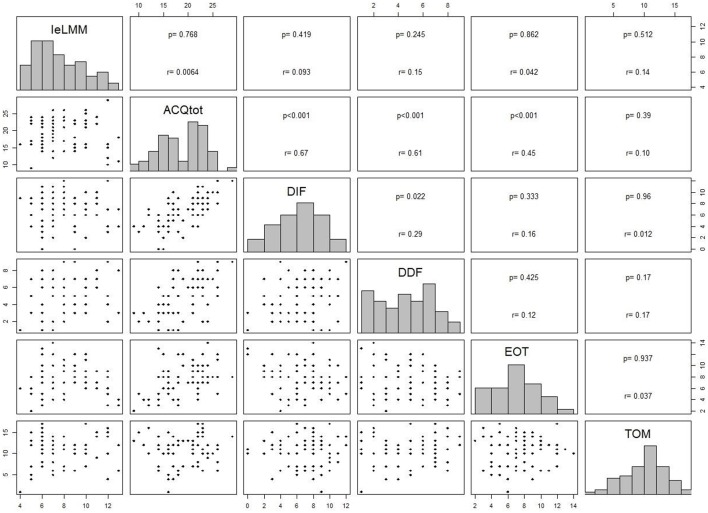
Correlation analysis between subscale scores of MCQ-C, AQC, and TOM.

**Figure 2:**
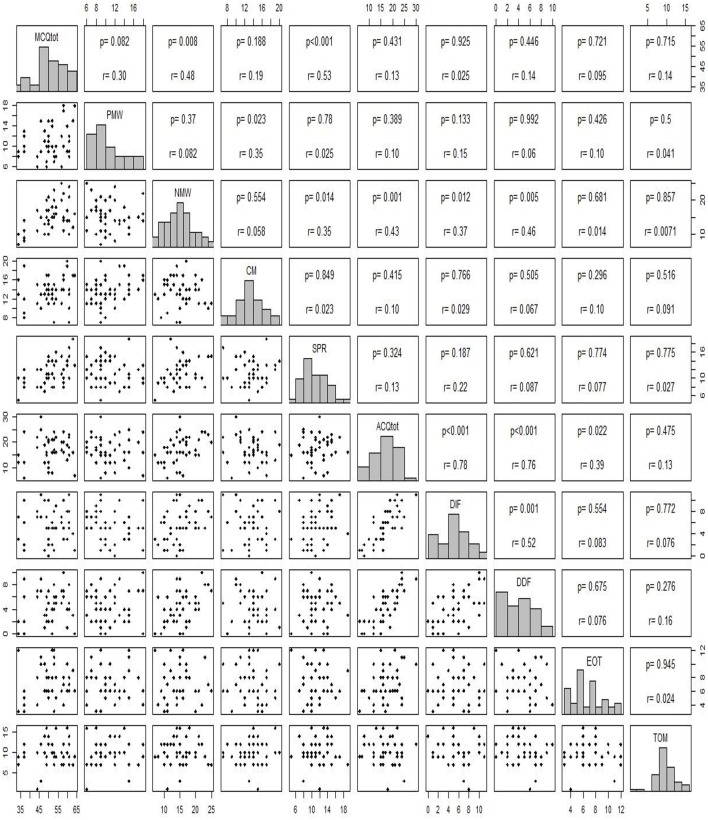
Correlation analysis between subscale score of IeLMM, AQC, and TOM.

In Table 3 we report the comparison of *IeLMM* and *MCQ-C* between EG and CG and between Males and Females. Table 4 shows the comparison of AQC and TOM of EG vs. CG, Male vs. Female and children aged 8–10 vs. pre-adolescents 11–13. In Table 5 there are within group results. In Figure 1 there are the correlation between *MCQ-C, AQC* and TOM and in Figure 2 between *IeLMM, AQC*, and TOM.

**Table 3:** EG vs. CG and male vs. female in the *IeLMM* and *MCQ-C*.

**Sub-scale**	**EG + CG Median (IQR)**	**EG Median (IQR)**	**CG Median (IQR)**	***p*-value**	**Male Median (IQR)**	**Female Median (IQR)**	***p*-value**
IeLMM	8 (6,10)	7 (6,9.7)	8 (7,10)	0.405	8 (6,9)	7 (6,10)	0.762
MCQ.tot	52 (44,58)	48 (41, 57.2)	53 (48,60)	0.143	48 (41,58)	53.5 (48.5, 58.5)	0.094
PMW	10 (8,12)	10 (8, 11.2)	10 (9,14)	0.236	10 (9,12)	10 (8, 12.2)	0.642
NMW	15 (12,19)	15 (10.7, 20.2)	16 (13,18)	0.466	14 (11,16)	17.5 (14, 20.2)	0.01^**^
CM	14 (11,16)	13 (11,16)	14 (12,16)	0.173	14 (11,16)	14 (11.7,16)	0.677
SPR	11 (9,14)	10 (9, 13.2)	12 (10,14)	0.33	11 (9,14)	11.5 (9.7,13)	0.823

** *p < 0.01*.

*CG, Control Group; CM, Cognitive Monitoring; EG, Experimental Group; IeLMM, Io e La Mia Mente; MCQ-C.tot, Metacognitions Questionnaire for Children total score; NMW, Negative Meta-Worry; PMW, Positive Meta-Worry; SPR, Superstition, Punishment, and Responsibility*.

**Table 4:** EG vs. CG, Male vs. Female, 8–10 vs. 11–13 years in *AQC* and TOM.

**Sub-scale**	**EG + CG Median (IQR)**	**EG Median (IQR)**	**CG Median (IQR)**	***p*-value**	**Male Median (IQR)**	**Female Median (IQR)**	***p*-value**	**8–10 years Median (IQR)**	**11–13 years Median (IQR)**	***p*-value**
AQC.tot	18 (15,22)	18.5 (16, 22.7)	17 (14,22)	0.063	17 (14.5,22)	19 (15,23)	0.173	21 (16,23)	17 (13,21)	0.007^**^
DIF	6 (4,8)	7 (5,9)	6 (3.2,8)	0.045^*^	6 (3,9)	7 (5,8)	0.625	7 (5,9)	6 (3,8)	0.024^*^
DDF	5 (3,7)	5 (4,7)	4 (2,6)	0.018^*^	4 (3,6.5)	5 (3,7)	0.181	5 (3,7)	4 (2,6)	0.128
EOT	7 (5,9)	7 (6,8)	8 (5,9)	0.523	7 (5,9)	8 (6,9)	0.485	8 (6,9)	7 (5,8)	0.19
TOM	10 (9,12)	10.5 (9,12)	10 (9,12)	0.985	10 (9,12)	11 (9,13)	0.208	11 (8.5,13)	10 (9,12)	0.185

* p < 0.05;

** *p < 0.01*.

*AQC.tot, Alexithymia Questionnaire for Children total; CG, Control Group; DDF, Difficulty Describing Feelings; DIF, Difficulty Identiying Feelings; EG, Experimental Group; EOT, Externally-Oriented Thinking; TOM, Theory of Mind*.

**Table 5:** Analysis within the EG.

**Sub-scale**	**Time since the beginning of pharmacologic treatment**				**Years from the onset**			**Associated symptoms**		
	** <6 Median (IQR)**	**(6, 12) Median (IQR)**	**>12 Median (IQR)**	***p*-value**	**≤3 years Median (IQR)**	**>3 years Median (IQR)**	***p*-value**	**Yes Median (IQR)**	**No Median (IQR)**	***p*-value**
IeLMM	7 (6,10)	7 (6,8.7)	8 (6.7,9.5)	0.805	7 (6,8.2)	7.5 (6, 10.7)	0.301	7 (6,8.5)	8 (6,10)	0.365
MCQ.tot	47 (40,57)	55 (46.5,59.7)	47 (41,56)	0.334	46 (43.2, 58.2)	50.5 (41,57)	0.97	49 (42,57.5)	49 (38.7, 57)	0.349
PMW	10 (9,10)	11 (10.2, 11.7)	9 (8,12)	0.342	10 (9,11)	9.5 (8, 11.7)	0.789	10 (8,11.5)	10.5 (9,11.5)	0.547
NMW	14 (11,20)	15 (12,18)	15 (10,21)	0.941	13.5 (10.2,18.5)	15 (11.2, 20.7)	0.634	15 (11,21)	14.5 (10.5, 15.5)	0.383
CM	11 (10,17)	16 (13.7,16)	13 (11,14)	0.356	13.5 (11,16)	13 (11,14)	0.774	13 (11,16)	12 (10.5,14)	0.497
SPR	12 (10,15)	12 (8.5,14.7)	10 (9,11)	0.454	10 (9,14)	10.5 (9,12)	0.909	10 (9,12)	12.5 (7.7, 14.7)	0.428
ACQ.tot	21 (16,22)	20 (16,23.5)	18 (16,22)	0.754	20.5 (16,23)	18 (16,22)	0.827	18 (16,22)	21 (16,23)	0.73
DIF	8 (6,10)	7 (5.7,9)	6 (3,8)	0.023	8 (5.2,9)	7 (4.7,8)	0.246	7 (5,9)	7 (5.5,9)	0.866
DDF	5 (3,7)	6 (5,7)	5 (4,7)	0.392	6 (3.2,7)	5 (4,7)	0.656	6 (4,7)	5 (3.5,7)	0.232
EOT	7 (6,8)	7 (4.7,8.5)	8 (6,10)	0.291	7 (5.2,8)	7.5 (6,10)	0.297	7 (5,8)	8 (6,10)	0.185
TOM	12 (9,14)	11 (7,12)	10 (9,12)	0.38	11 (8,13.5)	10 (9,12)	0.763	10 (9, 12.7)	11 (8,12)	0.751

*AQC.tot, Alexithymia Questionnaire for Children total score; CM, Cognitive Monitoring; DDF, Difficulty Describing Feelings; DIF, Difficulty Identifying Feelings; EG, Experimental Group; EOT, Externally-Oriented thinking; IeLMM, Io e La Mia Mente; MCQ-C.tot, Metacognitions Questionnaire for Children total score; NMW, Negative Meta-Worry; PMW, Positive Meta-Worry; SPR, Superstition, Punishment, and Responsibility; TOM, Theory of Mind*.

### Alexithymia

The *AQC* score*s* were significantly different between the experimental group and the control group in the subscales DDF and DIF (respectively, *p* = 0.04; *p* = 0.01), where the clinical group scored higher better in both subscales. Younger children (8–10) obtained higher scores in the total score (AQC.Tot) and in the DIF than pre-adolescents (11–13). There were no other significant differences among groups or between males and females. We observed no significant differences within the clinical group, for any of the variables. Finally, the total score of the *AQC* was statistically positive correlated as expected, with the three subscales DIF, DDF, EOT (*p* < 0.001). Furthermore, the DIF subscale correlated significantly, in a positive way, with the DDF subscale (*p* < 0.05). The total, DIF, and DDF subscales correlated positively with NMW (*p* < 0.001; *p* < 0.01; *p* = 0.005, respectively).

### Metacognition

No significant difference was identified neither between the children of the EG and those of the CG, nor between the males and the females. Furthermore, there were no significant differences within the EG. Even in the total score and in each subscale of the *MCQ-C* there were no significant differences between the migraineurs and the healthy pre-adolescents. The only significant difference was observed between males and females in the NMW subscale, with girls reporting higher scores (*p* = 0.01). The correlation analysis showed that the PMW scale positively correlated with the CM subscale (*p* = 0.02) and that NMW correlated positively with SPR (*p* = 0.01). Furthermore, there is a positive correlation between NMW subscale and all the subscales of ACQ, excepting for EOT. Visibly, each sub-scale of the *MCQ-C* positively correlated with the MCQ total score, except for CM.

### Theory of Mind

In the Domain of Social Perception of *Nepsy-II* there were no significant differences neither between the children in the EG and those in the CG, nor between males and females, or between children aged 8–10 and those aged 11–13 and within the EG. Moreover, the TOM test scores did not correlate with the score of any other test or subscale considered.

## Discussion

The first relevant result of this study is the association found among children with migraine and the higher presence of some alexithymic traits. In fact, the EG scored higher on DIF and DDF compared to the CG. This could suggest that children with MWoA have a greater difficulty identifying and describing their emotions than children without any disease. Although only a few publications in the literature have provided data on the association between headache (in particular migraine) and alexithymia in childhood, our results are in line with the evidence available on children with TTH (21, 28), on adolescents (8) and on adult patients (10–12). In addition, younger children (8–10 years), regardless of the group they belonged to, scored higher than the older children (11–13 years) in AQC.tot and DIF. Indeed, younger children could show more difficult to identifying and describe their feelings for a physiological immaturity of cognitive and emotional development as suggested by other studies (29–31).

The absence of statistical significance for all the analyses conducted within the experimental group could be explained by the fact that the variables we assessed (“Time since the beginning of pharmacological treatment,” “Years from the onset” and “Associated Symptoms”), do not affect the abilities of emotional recognition and expression. Probably the frequency and the intensity of the migraine attacks might have a greater impact (32).

There are not many studies that investigated the association between metacognition and TOM abilities and headache in childhood and adolescence (16). However, given the importance of beliefs and metacognitive processes for emotional regulation, it has been hypothesized that the cephalalgic subjects, in addition to having greater alexithymic traits, may also have lower metacognitive skills than healthy subjects. This hypothesis would be consistent with the slight cognitive dysfunction found in recent studies in migraine patients (33, 34). From the present research, no significant difference emerged in metacognitive abilities between the clinical group and the control group either in 8–10 year-old children nor in 11–13 pre-adolescents.

It is probable that despite the fact that the questionnaire *Io e La Mia Mente* is a reliable tool with a good internal consistency, it focuses more on the metacognitive strategies that help to improve learning abilities compared to the assessment of monitoring and understanding of interior personal experience. On the other hand, we can hypothesize that the life conditions of children with headache, do not influence the metacognitive abilities as much as those linked to alexithymia, and perhaps the differences in this area manifest themselves more at a later age. For this reason, we expected different results in older children in whom the *MCQ-C* more directly investigated the phenomenon of “worry,” a cognitive-emotional process often linked to negative outcomes and individual discomfort (24). Also in this case, there were no significant differences between the group of migraine patients and the control group. It is possible that the degree of awareness of the cognitive monitoring of one's thoughts is not necessarily linked to psychopathological outcomes and consequently the clinical and non-clinical groups do not differ even in the positive/negative beliefs reported.

However, we can assume that the significant difference emerged in the NMW subscale between male and female, where girls scored higher than boys is probably linked to the fact that generally during the transition to adolescence, and during adolescence itself, girls tend to have higher levels of anxiety, rumination, and depressive symptoms, as well as a negative attribution style compared to boys (24, 35, 36).

The observation that NMW positively correlates significantly with the AQC.tot, DIF, and DDF can be attributed to the fact that having greater negative metacognitive beliefs also results in greater difficulty in managing one's own feelings. Vice versa greater difficulty in recognizing and expressing one's emotions could lead to greater negative metacognitive beliefs. Finally, we were surprised by finding no difference between the CG and the EG in consideration of the close relationship between TOM and affective regulation. It would seem that TOM is independent from all the variables we studied and, moreover, that the migraine condition does not influence the development of this ability. In a future study it would be interesting to use a different tool for evaluating TOM.

In our sample we do not enrolled children and adolescents with chronic migraine. The chronicity process of pain, and in particular of migraine, in addition to an early onset may have an impact on mentalization and emotional awareness (37). It would be interesting to make a comparison between children and adolescents with chronic and episodic migraine and to detect any differences in mentalization and theory of mind abilities.

## Limitations and Future Prospective

One of the limitations of this research is the relatively low number of participants. A larger sample would allow to investigate and compare the variables excluded in this work such as: the intensity and the frequency of the migraine attacks, the familiarity, and the type of therapy. A second limitation is that the clinical sample includes a specific population of patients with MWoA who voluntarily attends a specialist center, and which could result in a *selection bias*. Finally, having used self-administered tests, the component of social desirability must be taken into account. Our plan is to compare children with migraine with aura, without aura and those with TTH to verify the impact of the diagnosis and of the different cephalalgic characteristics.

## Conclusion

Main results of this study showed no differences between children with MWoA and healthy subjects in Theory of Mind and in metacognitive abilities, but the experimental group has showed higher alexithymia traits compared to control group. Moreover, migraine characteristics do not seem to affect the psychological construct studied in this research.

The present research provides new and original data on the relationship between MWoA and alexithymia in childhood and pre-adolescence. This is a field where there are very few studies in literature and none has analyzed in depth the metacognitive and TOM aspects in these age groups. Our results on alexithymia are in line with the current theoretical assumptions and experimental evidences, and they contribute to confirm the existence of a statistically significant association between migraine and alexithymic traits in children and pre-adolescents. In addition, our results also allow to further validate the AQC, the only tool available to measure the construct of alexithymia in pediatric age. Furthermore, the identification of no difference between metacognitive and TOM abilities and MWoA between groups merits further investigation.

## Data Availability

The datasets generated for this study are available on request to the corresponding author.

## Ethics Statement

Written informed consent was obtained from all parent's subjects. The study was conformed to the ethical guidelines of the 1975 Declaration of Helsinki and was approved by the Ethical Committee of Policlinico Umberto I, Sapienza University of Rome (Prot. N 336/19).

## Author Contributions

VG, GN, AQ, and NF conceived and designed the study and they are responsible for data acquisition. DAF, AV, GT, DC, BB, RC, PV, FG, CP, and TG were responsible for critical revision of this manuscript. All authors approved the final version of this manuscript.

### Conflict of Interest Statement

The authors declare that the research was conducted in the absence of any commercial or financial relationships that could be construed as a potential conflict of interest.

## Abbreviations

AQC: Alexithymia Questionnaire for Children
CG: Control Group
CM: Cognitive Monitoring
DDF: Difficulty Describing Feelings
DIF: Difficulty Identifying Feelings
EG: Experimental Group
EOT: Externally-Oriented Thinking
IeLMM: Io e La Mia Mente
MCQ-C: Metacognitions Questionnaire for Children
MWoA: Migraine Without Aura
NMW: Negative Meta-Worry
PMW: Positive Meta-Worry
SPR: Superstition Punishment and Responsibility
TOM: Theory of Mind.
